# Flexible Compostable Composite Films Based on Plasticized Reprocessed PLA and Reinforced with Rice Husk and Rice Husk Biochar

**DOI:** 10.3390/polym18131637

**Published:** 2026-07-01

**Authors:** Sergio Gonzalez-Serrud, Ana Cristina González-Valoys, Marina P. Arrieta

**Affiliations:** 1 Departamento de Ciencias e Ingeniería de los Materiales, Facultad de Ingeniería Mecánica, Universidad Tecnológica de Panamá, Panama City 0819-07289, Panama; 2 Grupo de Investigación en Geoquímica Aplicada y Sostenibilidad (GeoAS), Universidad Tecnológica de Panamá, Panama City 0819-07289, Panama; ana.gonzalez1@utp.ac.pa; 3 Departamento de Ingeniería Química Industrial y del Medio Ambiente, Escuela Técnica Superior de Ingenieros Industriales, Universidad Politécnica de Madrid ETSII-UPM, C/José Gutiérrez Abascal, 28006 Madrid, Spain; 4 Facultad de Ingeniería Civil, Universidad Tecnológica de Panamá, Panama City 0819-07289, Panama; 5 Centro de Estudios Multidisciplinarios en Ciencias, Ingeniería y Tecnología (CEMCIT-AIP), Panama City 0819-07289, Panama; 6 Sistema Nacional de Investigación-Secretaria Nacional de Ciencia, Tecnología e Innovación (SNI-SENACYT), Clayton Ciudad del Saber Edif. 205, Panama City 0816-02852, Panama; 7 Grupo de Polímeros, Caracterización y Aplicaciones (POLCA), Universidad Politécnica de Madrid, C/José Gutiérrez Abascal, 28006 Madrid, Spain; 8 Centro de Investigación en Materiales Estructurales (CIME), Universidad Politécnica de Madrid, C/Profesor Aranguren 3, 28040 Madrid, Spain

**Keywords:** reprocessed PLA (rPLA), rice husk waste, biochar, acetyl tributyl citrate (ATBC), sustainable materials

## Abstract

In this study, the valorization of poly(lactic acid) (PLA) waste as well as rice husk into sustainable materials was explored. To simulate the industrial valorization of defective PLA parts, scraps and burrs, PLA was reprocessed (rPLA) by melt extrusion and further plasticized with 15 wt.% of acetyl tributyl citrate (ATBC) and reinforced with rice husk (RH) or rice husk biochar (RHB) in 1 or 3 wt.%. The melt flow index was determined to assess the effect of reprocessing and the addition of RH or RHB on the material degradation. The obtained films were characterized in terms of their structural, mechanical, and thermal behavior. The water-related behavior of the materials was evaluated by measuring the static water contact angle and the water vapor transmission rate (WVTR). Compostability was proposed as an end-of-life option, therefore disintegration under composting conditions was assessed. Reprocessing increased the MFI and slightly reduced the strength and the modulus, consistent with chain scission. ATBC facilitated the processability, improved the particles’ dispersion and provided ductility to the final materials. RH and RHB acted mainly as nucleating agents and strongly modified the surface wettability. A low RHB loading improved the WVTR, whereas a higher filler content and ATBC generally increased the WVTR. All the films were completely disintegrated within 18 to 21 days. These results show practical valorization routes to obtain rPLA films with tunable properties and to preserve the inherent composting disintegration of PLA.

## 1. Introduction

Plastic pollution and the growing demand for low-impact materials have accelerated the development of sustainable polymers and circular strategies that reduce reliance on fossil resources while improving end-of-life management [1,2]. In this context, poly(lactic acid) (PLA) has become one of the most widely adopted bio-based polymers due to its commercial maturity, processability, and inherent compostability under controlled conditions [3]. However, as PLA is mainly proposed for single-use applications, there is an increasing concern for the development of recycling strategies for PLA before throwing it away to be composted [3,4,5,6]. Moreover, replacing conventional plastics with PLA at the industrial scale still faces practical barriers related to performance, durability, and the variability introduced by real processing and reuse cycles [7]. Meanwhile, based on the European Commission objectives established for 2030, there is a need to increase the content of recycled material in all single-use applications as an essential prerequisite to its strategy to introduce plastics in a circular-economy concept [8].

Despite its advantages, PLA can exhibit limitations such as brittleness, sensitivity to hydrolysis and thermal history, and property losses during melt processing. These drawbacks become more critical when PLA is reprocessed (e.g., through additional extrusion cycles), where chain scission and molecular-weight reduction may alter the melt flow behavior and compromise the mechanical performance [9,10]. From a circular-economy perspective, mechanical reprocessing is attractive because it can extend the useful life of polymer streams and revalorize industrial scrap and burrs, which are materials with a well-known origin and that were not in contact with other waste such as that coming from a recycling stream, but additional strategies are often required to recover or tune their properties after reprocessing [7].

One effective approach to improve the flexibility and processability of PLA-based systems is plasticization. Acetyl tributyl citrate (ATBC) is a bio-based citrate plasticizer frequently used to increase PLA chain mobility, reduce brittleness, and enhance ductility in PLA-based films [11,12], making it relevant for flexible-film applications and for mitigating the performance losses associated with PLA reprocessing [9]. In this context, among other plasticizers, ATBC has been widely used for PLA plasticization due to the similarity in their solubility parameters (*δ*); the *δ*_PLA_ is between 19.5 MPa^1/2^ and 20.5 MPa^1/2^ [13] and the *δ*_ATBC_ is 20.2 MPa^1/2^ [14]. The plasticization of PLA with ATBC at 15 wt.% showed a good balance among the structure–property relationship of PLA–ATBC-based composites, while the overall migration levels assayed in a fatty food simulant showed low migration levels (below the migration limits required for food contact materials) [11]. Nevertheless, plasticization may also influence moisture sensitivity, thermal transitions, and degradation/disintegration behavior, which must be evaluated alongside the targeted mechanical improvements [15].

In parallel, circularity can be strengthened by integrating agro-industrial residues as functional fillers, converting waste streams into value-added resources. Rice husk (RH) is an abundant lignocellulosic byproduct whose incorporation into PLA can reduce material cost and environmental burden while potentially modifying stiffness, surface polarity, and water interactions [16]. A related, increasingly studied alternative is rice husk biochar (RHB), obtained via pyrolysis, which typically presents a more carbon-rich and less polar surface than untreated RH. Because RH and RHB differ in chemistry, porosity, and surface functionality, their effects on PLA/rPLA systems can diverge substantially, impacting dispersion, interfacial interactions, melt processability, thermal stability, and surface wettability [17].

Although PLA composites reinforced with natural fillers or biochar have been reported, most previous studies have focused on virgin PLA matrices or on single reinforcement systems considered separately, while fewer works have addressed mechanically reprocessed PLA as the polymer matrix [9,17,18,19]. In addition, recent reviews on PLA recycling have highlighted the growing interest in reprocessing strategies but also the limited number of studies specifically devoted to biofilled, reprocessed PLA systems [20]. In our previous study, we compared untreated rice husk (RH) and rice husk biochar (RHB) particles smaller than 500 µm to reinforce mechanically reprocessed PLA films. The specific aim was to determine whether both of the rice-derived fillers could be successfully incorporated into rPLA films while preserving their compostability and enabling a direct comparison of the effect of the filler nature on melt processability, thermomechanical behavior, surface-related properties, and disintegration under controlled composting conditions [21]. This first stage demonstrated that agro-industrial residues from the rice sector can be effectively integrated into rPLA films, thereby validating the feasibility of this circular strategy.

The present study moves beyond feasibility and introduces a second level of formulation aimed at improving the compatibility between the rPLA matrix and the rice husk particles. For this, a finer particle size fraction (<40 µm) for both RH and RHB was used, and a plasticizer was also added to increase the dispersion of such particles within the polymeric matrix. This reduction in particle size was not merely a processing modification but a deliberate strategy to increase the specific interfacial area between the filler and the rPLA matrix, favor a more homogeneous dispersion, and reduce the likelihood of large interfacial discontinuities typically associated with coarser particles in a polymeric matrix. In parallel, ATBC was incorporated to improve processability and flexibility, since the design of flexible compostable films requires not only the valorization of agricultural residues and reprocessed PLA but also tighter control over filler dispersion and matrix mobility. In this sense, this work extends the scientific contribution of the first one: while the 500 µm study established the viability of RH and RHB as sustainable reinforcements for rPLA films, the <40 µm study was conceived to optimize the structure–property relationships of these systems and to explore whether finer rice-derived particles, especially in the presence of ATBC, could provide a better balance among flexibility, water-related behavior, and compostable end-of-life performance. In this context, low filler contents of 1 and 3 wt.% were selected to compare the effect of RH and RHB within a controlled formulation window, allowing the influence of filler type and loading to be evaluated while avoiding the processing limitations and particle agglomeration commonly associated with higher natural-filler contents [12,15,22]. Similarly, ATBC was added at 15 wt.% on the basis of previous works on PLA–ATBC [11,14] and rPLA–ATBC blends [15]. Thus, the innovation of this work lies not only in adding a plasticizer, but also in demonstrating how particle-size engineering can be used as a formulation variable to tailor the performance of compostable rPLA-based films for agricultural applications.

## 2. Materials and Methods

### 2.1. Materials

Poly(lactic acid) (PLA) pellets (commercial grade Ingeo™ 2003D) were supplied by NatureWorks LLC (Minnetonka, MN, USA). According to the supplier, PLA presents a density of 1.24 g·cm^−3^ and a melt flow index (MFI) of 6 g/10 min, measured at 210 °C under a 2.16 kg load. Acetyl tributyl citrate (ATBC, 98% purity, Mw = 402 g·mol^−1^, Tm = −80 °C) was used as the plasticizer at 15 wt.% with respect to the polymeric matrix.

Poly(lactic acid) utilized in this study includes both virgin PLA and reprocessed PLA (rPLA), where PLA corresponds to extruded and further processed virgin PLA by compression molding into neat PLA films, while rPLA corresponds to PLA subjected to an additional melt-processing cycle to simulate the revalorization of defective industrial parts.

Rice husk (RH) was supplied by Cooperativa Avance R.L. (Los Olivos, Los Santos, Panama) and first sieved through a 40 μm mesh to obtain the fine fraction (RH < 40 μm), which was subsequently used to reinforce rPLA–ATBC blends according to the targeted formulations. The carbonization process was carried out by pyrolyzing whole (unsieved) rice husk in a fixed-bed reactor at 450 °C, using a heating rate of 10 °C·min^−1^ under an inert N_2_ flow of 12 Nl·min^−1^ in oxygen-free conditions. After pyrolysis, the resulting biochar was ground and sieved through a 40 µm mesh to obtain the fine fraction (RHB < 40 µm) used in the composite formulations. The pyrolysis temperature was selected based on prior studies reporting a balanced cost–benefit performance for rice-husk-derived biochar under comparable conditions [17]. Rice-husk-derived biochar (RHB) has been reported to enhance soil quality, largely because it can contribute to key nutrients including nitrogen, which is fundamental to soil fertility as it supports plant nutrient uptake and can help improve the soil’s capacity to retain moisture. Together, these effects may promote a more productive and sustainable agricultural system [23].

The films were produced using a set of formulations designed to evaluate the effects of both bio-based particulates (rice husk, RH, and rice husk biochar, RHB) and ATBC plasticization on reprocessed PLA (rPLA). The studied compositions are summarized in Table 1.

All blends were processed by melt extrusion using a 3DEVO Composer 350 extruder (3Devo, Utrecht, The Netherlands) followed by compression molding in a Mr. Hide Extracts WTRP-10T Rosin hot press (Mr. Hide Trading S.L., Tarragona, Spain) to obtain films. The filler contents of 1 and 3 wt.% were selected as representative low-loading levels based on previous studies on PLA-based composites containing biochar, rice-husk-derived fillers, or other biomass-derived particles [17,22,24,25]. Low filler contents allowed the tuning of crystallization, stiffness, barrier behavior, and surface properties while preserving processability, whereas higher filler loadings may promote particle agglomeration, interfacial defects, and loss of ductility [9], particularly in unplasticized PLA-reinforced systems [12].

#### 2.1.1. Steps to Manufacture the Materials

**Figure 1:**
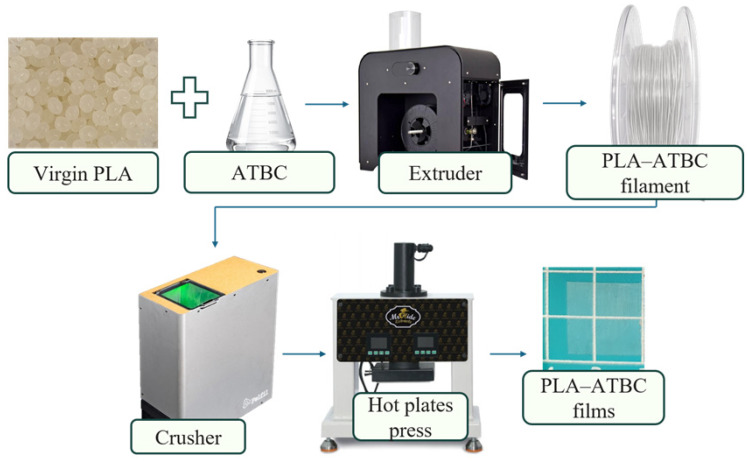
Methodology used to manufacture PLA–ATBC films.

**Figure 2:**
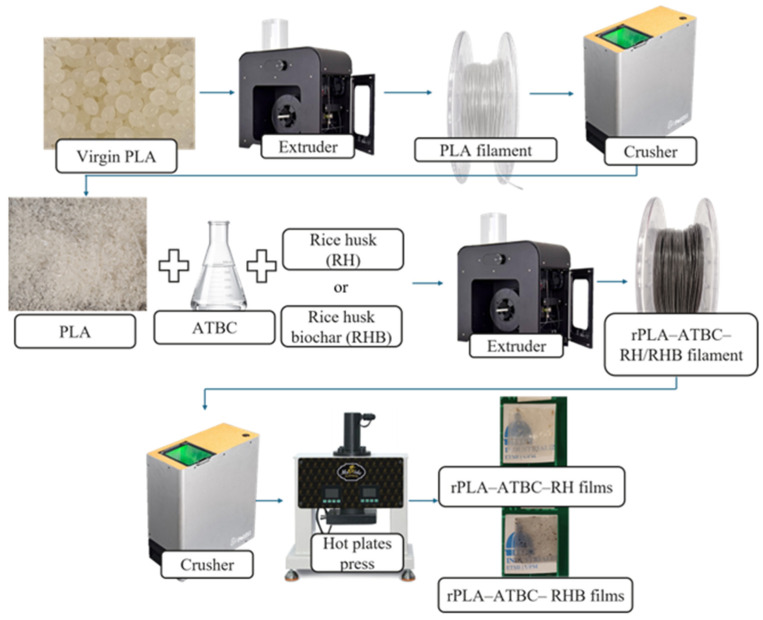
Methodology used to manufacture mechanically recycled plasticized composites films.

Figure 1 and Figure 2 schematically illustrate the manufacturing process of the PLA (Figure 1) and rPLA (Figure 2) films and the incorporation of the corresponding additives.

Figure 1 illustrates the processing methodology used to manufacture PLA-based films plasticized with 15 wt.% of ATBC. First, virgin PLA pellets were dried overnight at 60 °C in a vacuum oven (J.P. Selecta, Barcelona, Spain) to minimize hydrolytic degradation during melt processing. In the first step, the dried PLA pellets were mixed with ATBC in a sealed glass jar and further processed using a 3DEVO Composer 350 extruder (3Devo, Utrecht, The Netherlands) to produce PLA–ATBC filament. The temperature profile from the hopper to the die was set to 170, 185, 190, and 170 °C, and the screw speed was fixed at 4.5 rpm. The resulting PLA filament was then shredded using a Felfil Plastic Shredder+ Model 500 (Felfil, Turin, Italy) to obtain PLA–ATBC pellets, which were finally compression-molded in a Mr. Hide Extracts WTRP-10T Rosin hot-plate press (Tarragona, Spain) to produce the neat PLA–ATBC films.

Figure 2 illustrates the processing methodology used to manufacture the rPLA–ATBC-based films developed in this study. In the first stage, rPLA was produced following the procedure of Sepúlveda-Carter et al. [3]. For that, the dried virgin PLA pellets were processed in a 3DEVO Composer 350 extruder (3Devo, Utrecht, The Netherlands) to produce PLA filament. The temperature profile from the hopper to the die was set to 170, 185, 190, and 170 °C, and the screw speed was fixed at 4.5 rpm. The resulting PLA filament was then shredded using a Felfil Plastic Shredder+ Model 500 (Felfil, Turin, Italy) to obtain rPLA pellets suitable for blending with ATBC and/or the rice-based fillers simulating the mechanical recycling of industrial PLA waste. Therefore, in the second stage, these rPLA pellets were re-extruded using the same 3DEVO Composer 350 extruder to produce either (i) unfilled, reprocessed PLA filament plasticized with 15 wt.% ATBC (rPLA–ATBC) or (ii) plasticized composite filaments containing rice husk (RH) or rice husk biochar (RHB). For the composite systems, the shredded PLA was dry-mixed with 15 wt.% ATBC and 1 or 3 wt.% of RH or RHB particles (<40 μm) in a sealed glass jar to promote uniform filler distribution prior to the second extrusion step.

After reprocessing, the obtained filaments were shredded again using the Felfil Plastic Shredder+ Model 500 and subsequently compression-molded in a hot-plate press (Mr. Hide Extracts WTRP-10T Rosin press) to obtain the final films: rPLA–ATBC, rPLA–1%RH–ATBC, rPLA–3%RH–ATBC, rPLA–1%RHB–ATBC, and rPLA–3%RHB–ATBC.

#### 2.1.2. Press Conditions for Film Manufacturing

Polymeric films were produced by compression molding using a Mr. Hide Extracts WTRP-10T Rosin hot press set to 180 °C and a film mold with dimensions of 15 × 15 cm^2^. For each formulation, 2 g of material was placed in the mold and held under atmospheric pressure for 2 min to ensure complete melting. Films were then obtained through a stepwise pressure program intended to improve film uniformity and reduce entrapped air: 3 MPa for 1 min, 5 MPa for 1 min, and 10 MPa for 2 min. After the compression stage, the films were rapidly cooled to room temperature while maintaining atmospheric pressure. This quenching step helps preserve the polymer’s structural features and promotes a homogeneous microstructure, following the approach previously described [14,21].

### 2.2. Melt Flow Index

To assess the melt processability of PLA, rPLA, and the RH- and RHB-filled rPLA pellets, the melt flow index (MFI) was measured using a Metrotec MFI-100 instrument (Techlab Systems, Lezo, Spain). Tests were conducted at 170 °C with a 2.16 kg applied load. For each formulation, six sequential determinations were performed, and the extrudate mass was collected and recorded over 15 s for each run, according to UNE-EN ISO 1133-1 [26].

### 2.3. Attenuated Total Reflectance—Fourier Transform Infrared Spectroscopy (ATR-FTIR)

Chemical structure and functional groups were characterized by Fourier Transform Infrared (FTIR) spectroscopy, which probes molecular vibrations through the absorption of infrared radiation. Spectroscopic examination was conducted utilizing a 4X FT/IR spectrometer manufactured by Jasco Corporation, headquartered in Hachioji, Tokyo, Japan. Absorbance measurements were performed across a wavelength span ranging from 4000 cm^−1^ to 400 cm^−1^, employing 24 scan repetitions and a resolution of 4 cm^−1^ [7].

### 2.4. Scanning Electron Microscopy (SEM)

Cross-sectional microstructures of the films were analyzed by field-emission scanning electron microscopy (FESEM) on cryogenically fractured surfaces. Samples were first immersed in liquid nitrogen (N_2_) and then fractured to expose the cross-section. Prior to imaging, the fracture surfaces were sputter-coated with a gold–palladium (Au/Pd) alloy to ensure adequate electrical conductivity [3].

### 2.5. Mechanical Properties

Tensile behavior was evaluated at room temperature using an Autograph AGS-10 Series universal testing machine equipped with a 100 N load cell (Shimadzu Corporation, Kyoto, Japan). Testing followed UNE-EN ISO 527-4 [27]. Rectangular specimens (5 mm × 30 mm) were tested using an initial grip separation of 20 mm and a crosshead speed of 5 mm·min^−1^. From the resulting stress–strain curves, strain at break (εb), tensile modulus (Et), and tensile strength (σt) were obtained. Reported values correspond to the average of six replicates per formulation [9].

### 2.6. Differential Scanning Calorimetry (DSC)

Thermal transitions of PLA, rPLA, and the selected RH- and RHB-reinforced rPLA films (1 and 3 wt.%) were analyzed by differential scanning calorimetry using a SETLINE DSC from SETARAM (Caluire, France). The thermal program consisted of three stages: (i) a first heating from 25 to 200 °C to erase prior thermal history, (ii) cooling to 0 °C, and (iii) a second heating up to 240 °C. All steps were performed at 10 °C·min^−1^ under nitrogen at 30 mL·min^−1^. Samples (approximately 5–8 mg) were sealed in 40 μL aluminum pans. The degree of crystallinity (X_c_) was calculated from the melting and cold-crystallization enthalpies (ΔH_m_ and ΔH_cc_), using ΔH_0m_ = 93 J·g^−1^ as the melting enthalpy of fully crystalline PLA [3,28], with the Equation (1).

(1) $$X_{c}\left(\%\right)=\left[\frac{\Delta H_{m}-\Delta H_{cc}}{\Delta H_{0m}\cdot \left(1-w\right)}\right]\times 100\%$$

### 2.7. Thermogravimetric Analysis (TGA)

Thermogravimetric behavior was evaluated by dynamic TGA using a TGA 2050 Thermogravimetric Analyzer, SETARAM (Caluire, France). For each formulation, a representative specimen (≈10 mg) was placed in a platinum pan and heated from 40 to 800 °C at a constant rate of 10 °C·min^−1^ under a nitrogen atmosphere. During the run, the instrument continuously monitored sample mass as a function of temperature, enabling the determination of thermal stability and decomposition profiles [15,29].

### 2.8. Static Contact Angle Measurements

Film surface wettability was assessed via static water contact angle measurements using a goniometer (Ossila BV, Leiden, The Netherlands) equipped with a camera and Ossila Software version 4.1.4. Distilled-water droplets (≈10 μL) were deposited onto the film surfaces using a syringe, and contact angles were recorded for approximately ten measurements per sample, placing films in randomized positions to improve representativeness in accordance with the ASTM D5946-17 [30].

### 2.9. Water Vapor Transmission Rate

The water vapor transmission rate (WVTR) of the bio-based films was determined by a gravimetric method using silica gel as desiccant. Film specimens with an exposed area of 10 cm^2^ were sealed onto permeability cups containing 2 g of silica gel. The cups were placed inside a desiccator kept at 23 ± 1 °C and approximately 90% relative humidity, which was generated using a saturated potassium nitrate (KNO_3_) solution. The mass of each assembly was measured hourly for 6 h. WVTR values (g·day^−1^·cm^−2^) were calculated from the mass increase over time, where m_t_ corresponds to the cup mass at time t, m_0_ is the initial mass, and A is the exposed film area. Results were normalized to a reference film thickness of 100 μm, following the procedures reported in the literature and in the UNE-EN ISO 53097 standard [31].

### 2.10. Disintegration Under Composting Conditions

Disintegration under composting conditions was assessed at laboratory scale according to UNE-EN ISO 20200 [32]. Film pieces (15 × 15 mm) were buried at approximately 6 cm depth in perforated plastic containers filled with a synthetic composting medium. The compost mixture (wet basis) consisted of 10% compost (Mantillo, Granada, Spain), 30% rabbit feed, 10% starch, 5% sugar, 1% urea, 4% corn oil, and 40% sawdust, and water was added to reach approximately 50 wt.% moisture content. The containers were incubated under aerobic conditions at 58 °C. Samples were removed after 1, 4, 7, 9, 11, 14, and 21 days to track the evolution of disintegration. At each sampling time, specimens were photographed to provide a qualitative record of physical fragmentation and surface deterioration over time [5,15].

### 2.11. Statistical Analysis

Statistical analyses were performed using Python 3.13.12. Analysis of variance (ANOVA) was used to evaluate the data, and Fisher’s least significant difference (LSD) test was applied to determine differences among samples. Statistical significance was established at *p* < 0.05.

## 3. Results and Discussion

### 3.1. Melt Flow Index

**Figure 3:**
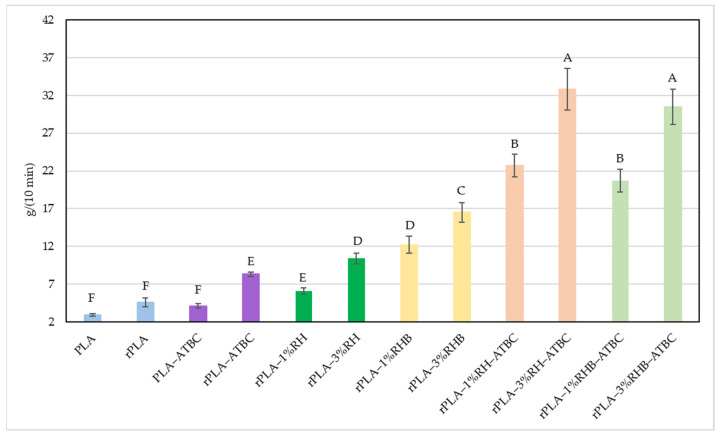
Determination of the MFI of PLA, rPLA, and rPLA-based composite pellets in bulk (170 °C, 2.16 kg). Different letters (A–F) indicate statistically significant differences among the formulations (*p* < 0.05).

The melt flow index (MFI) results, measured at 170 °C under a 2.16 kg load, are presented in Figure 3.

The MFI temperature of 170 °C and a loading of 2.16 kg wer selected considering the plasticizing effect of ATBC, which increases polymer-chain mobility and may lead to excessively high melt-flow values at higher testing temperatures. The results showed that mechanical reprocessing clearly increased melt flowability, with the MFI increasing from 2.9 ± 0.1 g/10 min for neat PLA to 4.6 ± 0.4 g/10 min for rPLA. Commercial PLA grades tested under the same conditions have been reported to exhibit MFI values of approximately 3.56 g/10 min [33,34], supporting the range obtained in the present study. This increase was consistent with a reduction in molecular weight driven by chain scission during the additional extrusion cycle, which decreased the melt viscosity and enhanced the melt flow. Accordingly, the MFI results confirmed that the thermomechanical history modified the processability, yielding a higher flow response than that of neat PLA [7,35].

The incorporation of less than 40 µm RH and RHB without a plasticizer showed a loading-dependent effect: for 1 wt.% rPLA–1%RH: 4.1 ± 0.2; and for rPLA–1%RHB: 3.7 ± 0.3 g/10 min). The MFI slightly decreased relative to rPLA, suggesting flow restriction due to the presence of particles and matrix–filler interactions that increased viscous resistance. However, when the loading increased to 3 wt.%, the MFI rose markedly (rPLA–3%RH: 8.3 ± 0.2; rPLA–3%RHB: 10.4 ± 0.5 g/10 min), indicating that processing-induced degradation in the presence of particulates dominated over the typical filler effect, thereby reducing the apparent melt viscosity [17].

As expected, the ATBC plasticizer increased the MFI in all the formulations. PLA–ATBC reached 12.2 ± 0.8 g/10 min and rPLA–ATBC 16.5 ± 0.9 g/10 min, reflecting the viscosity reduction associated with plasticization and the increase in segmental mobility. In the plasticized composites, the MFI increased even further (rPLA–1%RH–ATBC: 22.7 ± 1.1; rPLA–3%RH–ATBC: 32.8 ± 2.0; rPLA–1%RHB–ATBC: 20.7 ± 1.1; rPLA–%3 RHB–ATBC: 30.5 ± 1.6 g/10 min), evidencing a combined effect of plasticization and a melt that became more prone to flow under load [15]. Overall, the results showed that processability was primarily governed by ATBC, whereas the addition of RH/RHB modulated the response depending on the filler type and content, with particularly pronounced increases at 3 wt.% in the presence of the plasticizer.

### 3.2. Attenuated Total Reflectance—Fourier Transform Infrared Spectroscopy (ATR-FTIR)

**Figure 4:**
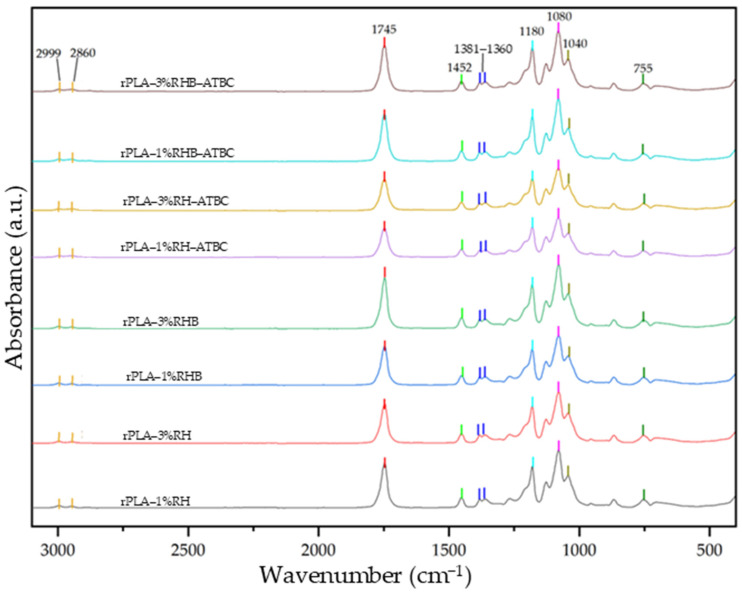
FTIR spectral analysis of rPLA-based films and their composites.

Figure 4 displays the FTIR spectra obtained for all the films.

The ATR-FTIR spectrum of the PLA film shows the characteristic absorption bands that are typically used to assess the polymer’s chemical structure and possible interactions with additives. Interpreting these bands is important to confirm the structural integrity of PLA. A strong signal at 1744 cm^−1^, assigned to the C=O stretching of the ester groups in lactide units, is one of the most prominent features of PLA spectra [3]. The band near 1450 cm^−1^ is associated with CH_3_ bending vibrations, while the region around 1180 cm^−1^ corresponds to C–O stretching of the ester linkages within the PLA backbone [15]. In addition, the peak at 1079 cm^−1^ is attributed to C–O–C stretching, related to the glycolic-type linkages that form part of the polymer chain [36].

The 3000–2860 cm^−1^ interval is dominated by C–H stretching vibrations. A broad contribution close to 3000 cm^−1^, linked to cyclohexene-type groups, partially overlaps these C–H bands. The spectra also include deformation features of C–H groups, with signals near 1380 cm^−1^ and 1360 cm^−1^ assigned to symmetric and asymmetric modes, respectively, together with the main ester carbonyl band at 1744 cm^−1^ and the methyl-related band at 1450 cm^−1^ [3].

Across all the formulations, both the composites and rPLA retained the typical PLA absorption pattern. After reprocessing and filler incorporation, no relevant peak displacements or additional bands were observed, indicating that the PLA backbone remained chemically preserved within the sensitivity limits of ATR-FTIR.

### 3.3. Scanning Electron Microscopy (SEM)

**Figure 5:**
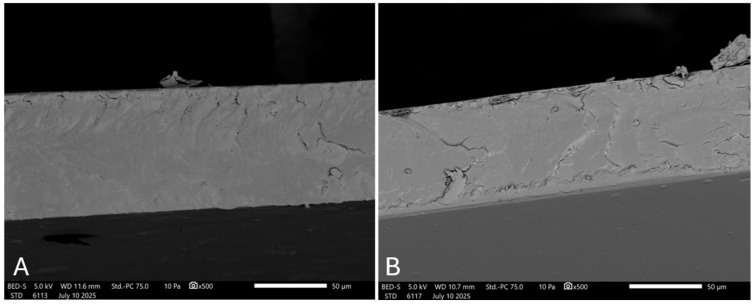
FE-SEM observations of films: (**A**) PLA–ATBC and (**B**) rPLA–ATBC.

**Figure 6:**
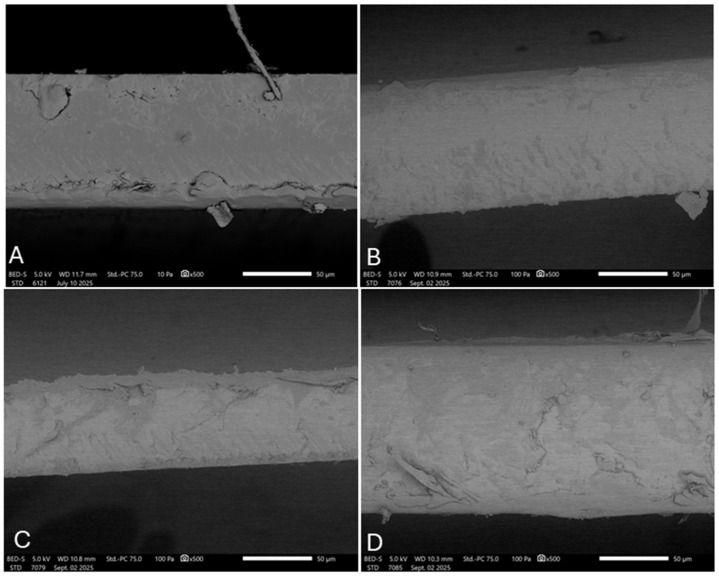
FE-SEM observations of films: (**A**) rPLA–1% RH, (**B**) rPLA–3%RH, (**C**) rPLA–1%RHB, and (**D**) rPLA–3% RHB.

**Figure 7:**
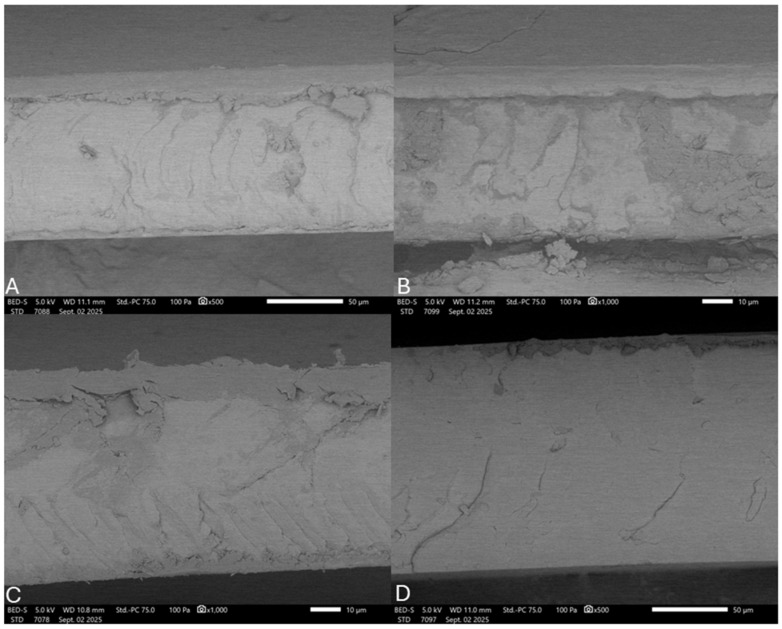
FE-SEM observations of films: (**A**) rPLA–1% RH—ATBC, (**B**) rPLA–3% RH—ATBC, (**C**) rPLA–1% RHB—ATBC, and (**D**) rPLA–3% RHB—ATBC.

Figure 5, Figure 6 and Figure 7 show representative SEM micrographs of the cryo-fractured cross-sections, illustrating the characteristic morphology of each formulation analyzed.

As reported in a previous work [16], the SEM micrographs of the PLA film showed a uniform and continuous fracture surface, with a predominantly smooth topography and no clear evidence of tearing or void formation. This morphology was associated with the brittle fracture behavior typically observed in unmodified semicrystalline PLA matrices [10,16]. In contrast, the rPLA film exhibited a rougher fracture surface while maintaining overall continuity within the observed section. Compared with PLA, rPLA showed a more heterogeneous texture, with scattered microcracks and localized surface relief, which was previously attributed to the cumulative effects of thermomechanical reprocessing, including chain scission and microstructural changes that can increase fracture irregularity [16]. In the SEM micrographs of the PLA–ATBC and rPLA–ATBC formulations analyzed in the present study, indications of a comparatively more ductile fracture response were observed (Figure 5A,B), with more cohesive and homogeneous surfaces and no apparent signs of phase separation. This behavior suggests good compatibility and effective plasticizer dispersion within the PLA/rPLA matrices, in agreement with the thermal response previously observed by the DSC and TGA analyses.

In the rPLA films reinforced with RH or RHB (Figure 6A–D), no large cavities, voids, phase-separated domains, or significant structural defects were observed at the analyzed magnification. This suggests that the incorporation of RH or RHB did not produce major morphological discontinuities that could compromise the structural integrity of the films, as was previously observed by Agüero et al. [15].

In contrast, the ATBC-plasticized films (Figure 7A–D) exhibited features consistent with higher plastic deformation and comparatively more ductile fracture patterns, which can be attributed to an effective distribution of the plasticizer within the rPLA–RH/RHB matrix [14,37]. This trend agrees with the thermal response previously observed by the DSC and TGA analyses and supports the effectiveness of ATBC in increasing segmental mobility and improving elongation at break in rPLA-based formulations.

### 3.4. Mechanical Properties

**Figure 8:**
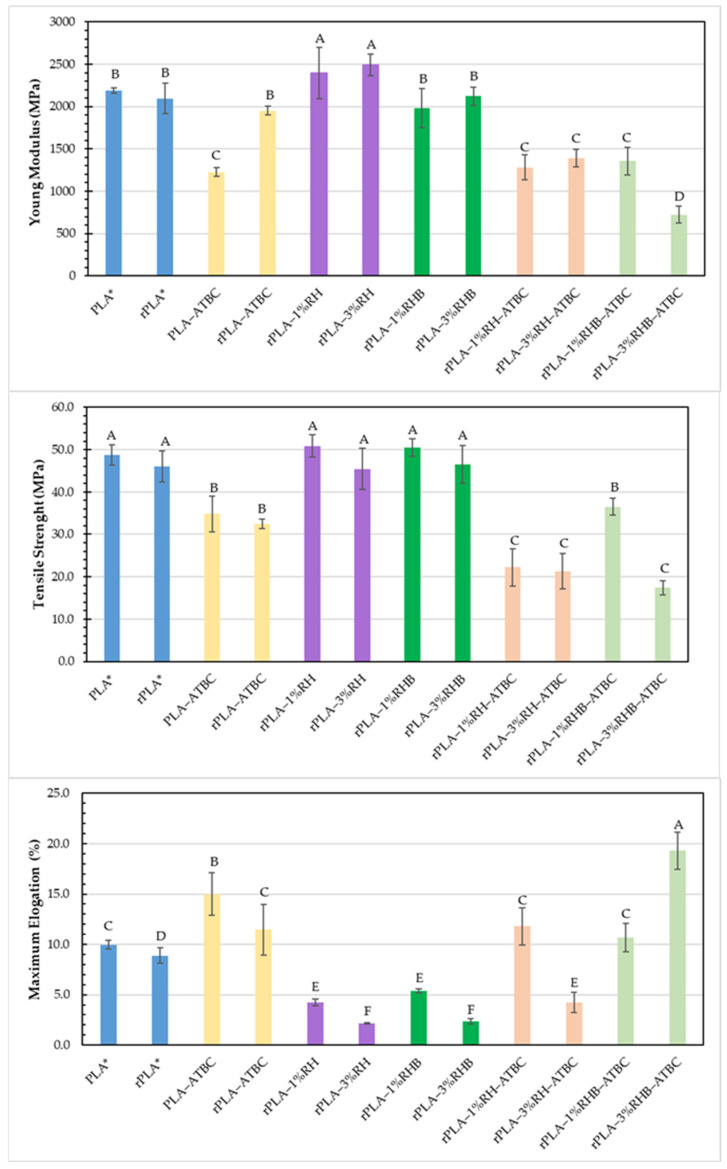
Tensile test average values of the biobased films. Different letters (A–F) indicate statistically significant differences among the formulations (*p* < 0.05). * The mechanical property results corresponding to PLA and rPLA were taken from the previous work of Gonzalez-Serrud et al. (2026) [16] and are included here for comparison purposes.

The tensile properties of the PLA based films, including the RH- and RHB-reinforced composites with and without ATBC, are summarized in Figure 8. Young’s modulus (Et), tensile strength (σt), and elongation at break (εb) were used to describe the mechanical response of the formulations.

Although the mean value of the tensile strength, σt, somewhat decreased from PLA (48.70 ± 2.38 MPa) to rPLA (46.05 ± 3.71 MPa), both the formulations belong to the same statistical group (A), indicating no significant difference in ultimate tensile strength at *p* < 0.05 [16]. In practical terms, the additional melt-processing step introduced a downward trend consistent with chain scission, but the magnitude of the change remained within experimental scatter and did not translate into a measurable loss of σt. This strength retention with early signs of degradation is consistent with recent reprocessing studies reporting that limited reprocessing can preserve tensile strength while other properties (especially ductility) are more sensitive to molecular-weight reduction [7,35].

For the unplasticized composites, the σt remained statistically comparable to PLA/rPLA (group A) at both 1 and 3 wt.% RH and RHB (45–51 MPa), suggesting that low filler loadings did not introduce critical defects or severe stress concentration. Similar behavior has been described for low-biochar PLA systems, where small additions can preserve strength when dispersion is adequate, whereas higher loadings may become defect-driven [17,24].

By contrast, the plasticized systems showed the expected strength penalty: PLA–ATBC and rPLA–ATBC dropped to group B (32–35 MPa), and the RH–ATBC formulations further decreased to group C (21–22 MPa). This agrees with the well-established trade-off of citrate plasticization in PLA, where increased chain mobility improves deformability but reduces load-bearing capacity [38,39]. Notably, rPLA–1%RHB–ATBC (36.5 ± 2.0 MPa, group B) retained a higher σt than RH–ATBC, indicating a more favorable balance between plasticization and reinforcement for that specific combination.

PLA (2189 ± 32 MPa) and rPLA (2096 ± 178 MPa) share the same letter (B) for the Young’s modulus (Et), again indicating no significant stiffness loss after reprocessing, despite the lower mean value [16]. This is consistent with reports showing that stiffness can be comparatively less sensitive than ductility to moderate molecular-weight reductions, particularly when crystallinity changes counterbalance chain scission effects [7].

RH reinforcement produced the clearest stiffening: rPLA–1%RH and rPLA–3%RH were in group A (2400–2490 MPa), significantly higher than PLA/rPLA (group B), consistent with the rigid lignocellulosic nature of RH and its nucleating contribution to the PLA matrix. Similar modulus gains are frequently observed in PLA systems containing lignocellulosic fillers when the particles are well distributed and the load transfer is effective [40,41].

In contrast, RHB-filled rPLA remained in group B (1980–2120 MPa), suggesting that, at 1–3 wt.% and without ATBC, the biochar did not significantly stiffen the matrix relative to PLA/rPLA. This outcome is consistent with the dependence of biochar reinforcement efficiency on biochar type, porosity/ash content, and interface quality [17,24]. Plasticization reduced stiffness most clearly in PLA–ATBC (group C, 1226 ± 54 MPa), while the lowest modulus occurred for rPLA–3%RHB–ATBC (group D, 723 ± 99 MPa), reflecting a highly softened network where both plasticization and filler-related microstructural discontinuities likely contribute.

For the elongation at break (εb), the impact of reprocessing becomes statistically evident: PLA (9.97 ± 0.42%, group C) and rPLA (8.89 ± 0.76%, group D) do not share letters, indicating a significant reduction in ductility after reprocessing [16]. This is a common signature of chain scission in PLA, where reduced entanglement density and shorter chains limit plastic deformation before fracture [35].

ATBC increased εb, with PLA–ATBC reaching 15.0 ± 2.1% (group B). The highest ductility was observed for rPLA–3%RHB–ATBC (19.3 ± 1.8%, group A), demonstrating that, in this formulation, plasticization dominated the failure response and enabled extensive deformation. This is in line with recent studies showing that citrate plasticizers can markedly raise PLA ductility, although the final outcome depends on the additive compatibility and microstructure [38,39].

However, the effect was filler-dependent: RH–ATBC at 3 wt.% exhibited a very low εb (4.24 ± 0.99%, group E), indicating that increasing the RH content in a plasticized matrix can still promote premature failure, likely via interfacial debonding or particle-driven strain localization—behaviors widely reported for hydrophilic lignocellulosic particulates in PLA when interfacial adhesion is not sufficiently strengthened [40].

### 3.5. Differential Scanning Calorimetry (DSC)

The thermal parameters obtained, including the glass transition temperature (T_g_), the cold crystallization temperature (T_cc_), the melting temperature (T_m_), the associated enthalpies (ΔH_cc_ and ΔH_m_), and the degree of crystallinity (X_c_), are summarized in Table 2.

The DSC results previously obtained by Gonzalez-Serrud et al. (2026) [16] indicated that reprocessing had a limited effect on the glass transition temperature but noticeably promoted crystallization. T_g_ remained essentially constant in rPLA with respect to neat PLA (≈59 °C), whereas T_cc_ shifted to a lower temperature (from 119.9 to 115.2 °C) and crystallinity (X_c_) increased (from 8.0 to 10.0%), which was consistent with a matrix containing shorter chains and a higher concentration of chain ends that crystallized more readily during the thermal cycle [16]. In this sense, recent reprocessing studies have reported minor changes in T_g_ but a tendency toward lower cold-crystallization temperatures and higher crystallinity as thermal history and molecular-weight reduction accumulate [3]. The incorporation of ATBC produced the most pronounced thermal shift: T_g_ decreased by approximately 20 °C (PLA–ATBC: 39.1 °C; rPLA–ATBC: 37.9 °C), and the crystallization window moved to a lower temperature (notably, rPLA–ATBC showed T_cc_ = 95.8 °C), which was consistent with citrate plasticization increasing free volume and segmental mobility [39,42].

By contrast, adding RH or RHB into the rPLA matrix increased the crystallinity (Xc ≈ 23–29%), while the T_g_ remained around 59 °C and the T_m_ stayed nearly constant (149–150 °C), indicating that the fillers acted mainly as nucleating agents rather than modifying the crystalline phase itself. The marked increases in ΔH_m_ and X_c_ were consistent with heterogeneous nucleation and faster crystal development in the PLA-based composites containing biomass-derived particles, including biochar, as recently reported [24]. The plasticized composites showed a filler-dependent response: rPLA with 1% and 3% RH–ATBC formulations exhibited low X_c_ values (0.9–5.0%), whereas rPLA with 1% and 3% RHB–ATBC formulations reached the highest crystallinity (≈30%).

This divergence suggested that, in the presence of ATBC, the biochar surface provided more effective nucleation sites, translating the increased chain mobility into crystallization during the DSC cycle, whereas the RH-containing systems did not convert plasticization into a comparable crystallinity gain. The strong dependence of PLA crystallization on both plasticization and nucleating characteristics has been emphasized in recent studies, which showed that the final crystallization response arises from the interplay between increased segmental mobility induced by the plasticizer and the heterogeneous nucleation ability of the filler [42,43,44]. In this regard, Blázquez-Blázquez et al. (2024) reported that the crystallization behavior of PLA and its composites was markedly affected by the combined action of the plasticizer and the mesoporous MCM particles, evidencing that the crystallization-promoting effect of plasticization is highly sensitive to the nature of the nucleating phase [42].

### 3.6. Thermogravimetric Analysis (TGA)

**Figure 9:**
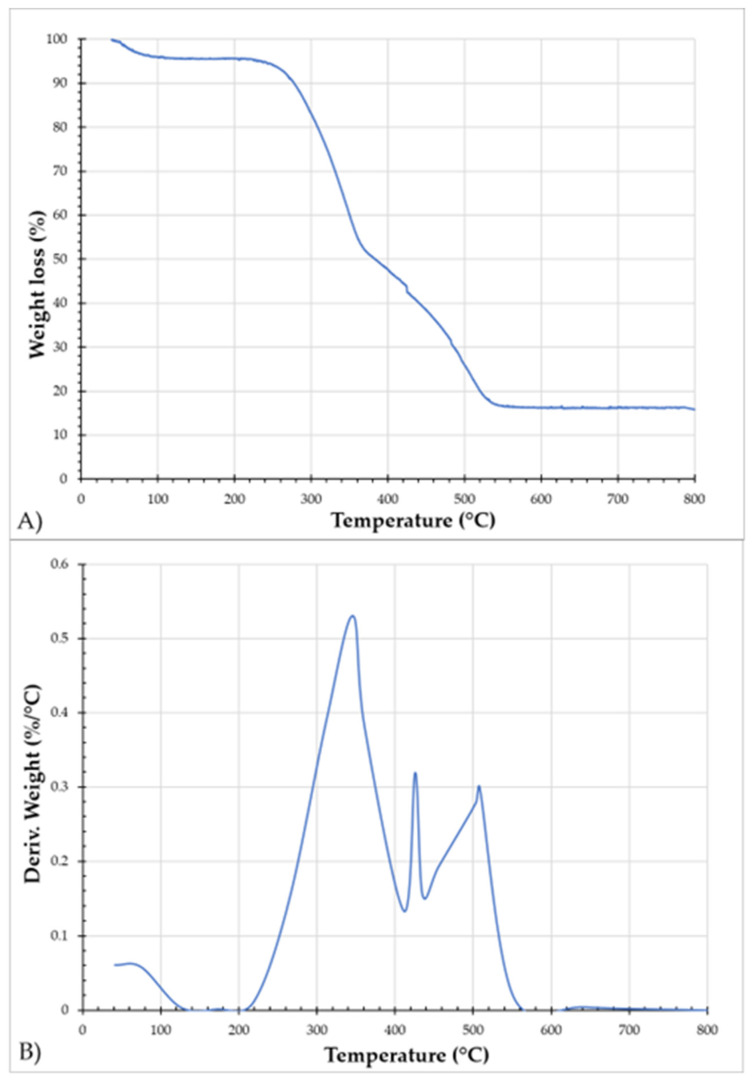
(**A**) Dynamic thermogravimetric analysis (TGA) curve and (**B**) derivative thermogravimetric (DTG) curve of rice husk (RH).

Figure 9 and Table 3 summarize the thermogravimetric data obtained for rice husk (RH).

The thermogravimetric analysis of untreated rice husk (RH) revealed a multi-step thermal degradation behavior, which is characteristic of lignocellulosic biomass due to the overlapping decomposition of hemicellulose, cellulose, lignin, extractives, and inorganic constituents [45]. The temperature corresponding to 5% mass loss was 234.3 °C, whereas the temperature associated with 10% mass loss was 277.9 °C. These results indicate that RH exhibits adequate thermal stability below approximately 230 °C, which is particularly relevant for its incorporation into reprocessed PLA matrices, considering that the processing temperatures used for the film fabrication were below this range.

The DTG curve showed three main degradation events. The first peak was observed at 346.2 °C, with a cumulative mass loss of 37.7%. This event can be mainly attributed to the thermal degradation of the polysaccharide fraction of the biomass, particularly hemicellulose and cellulose, which are known to decompose predominantly in the intermediate temperature range during biomass pyrolysis [45,46]. The second peak appeared at 426.1 °C, with a cumulative mass loss of 57.7%, and may be associated with the progressive degradation of more thermally stable fractions, such as lignin, as well as with carbonaceous structures formed during the initial decomposition stages. Lignin degradation generally occurs over a broader temperature range because of its complex aromatic structure and heterogeneous bonding network [47]. Finally, the third peak was detected at 508.7 °C, with a cumulative mass loss of 76.5%, indicating the degradation of residual organic or carbonaceous fractions with higher thermal stability.

At the end of the test, a final residue of 15.8% was obtained, corresponding to non-volatile solids. This residue can be attributed to the mineral fraction of rice husk, which is commonly associated with its relatively high silica content [48].

**Figure 10:**
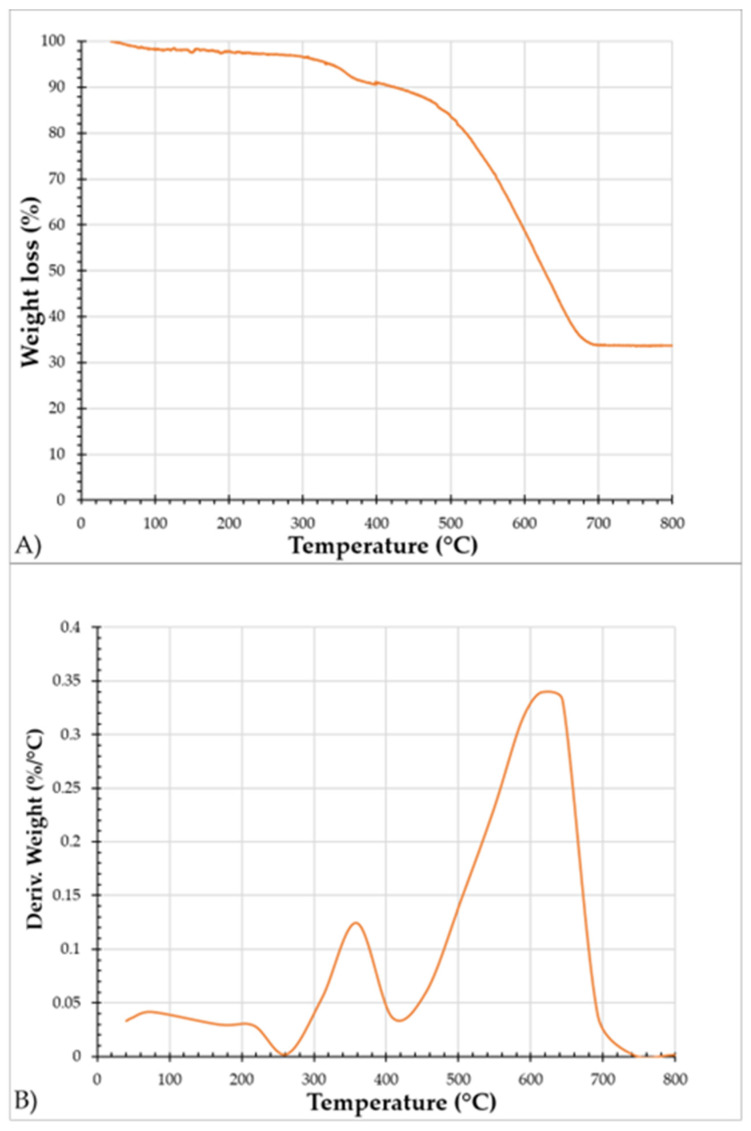
(**A**) Dynamic TGA and (**B**) its derivative (DTG) curve for rice husk biochar.

Figure 10 and Table 4 summarize the thermogravimetric data obtained for rice husk biochar (RHB).

The TGA results of rice husk biochar (RHB) revealed a remarkable increase in thermal stability compared with untreated rice husk. The temperature corresponding to 5% mass loss was 335.6 °C, while the temperature associated with 10% mass loss was 422.7 °C. These values indicate that RHB exhibits a high initial thermal resistance, which is advantageous for its incorporation into reprocessed PLA matrices, considering that the processing temperatures used for the film fabrication were far below this thermal degradation range. This improvement in thermal stability can be attributed to the carbonization process, which reduces volatile matter and promotes the formation of more thermally stable carbon-rich structures [49].

The DTG curve showed two main mass-loss events. The first peak was recorded at 359.2 °C, with a cumulative mass loss of only 7.1%. This event may be attributed to the degradation of remaining organic fractions, surface functional groups, or less stable components that were not completely removed during pyrolysis. The low cumulative mass loss observed at this stage confirms that carbonization substantially reduced the content of volatile compounds and easily degradable lignocellulosic components, as commonly reported for biochars produced at elevated pyrolysis temperatures [49].

The second DTG peak was observed at 613.0 °C, with a cumulative mass loss of 45.6%. This event may be associated with the thermal degradation or oxidation of more stable carbonaceous structures and recalcitrant organic fractions generated during pyrolysis. The appearance of this peak at a high temperature confirms that RHB requires a higher thermal energy to undergo significant mass loss, evidencing its enhanced thermal stability compared with non-carbonized rice husk. This behavior is consistent with the progressive aromatization and structural condensation of biomass-derived chars during thermal conversion [50].

The final residue, corresponding to the non-volatile solid fraction, was 33.7%. This value indicates that RHB retains a considerable amount of residual material after heating, which can be attributed to stable carbonaceous solids and the intrinsic mineral fraction of rice husk. In particular, rice-husk-derived biochars are known to contain a significant inorganic fraction, mainly associated with silica-rich ash [49].

Table 5 below summarizes the thermal parameters obtained from the thermogravimetric analysis (TGA).

The TGA results of Gonzalez-Serrud et al. (2026) showed that PLA exhibited higher thermal stability, with T_5_% = 323.4 °C and T_max_ = 371.2 °C, whereas rPLA displayed lower values (T_5_% = 318.2 °C; T_max_ = 364.7 °C), which was consistent with the reduction in the viscosity molecular weight associated with reprocessing (chain scission and a higher concentration of reactive chain ends) [16]. Comparable values were reported by Sepúlveda-Carter et al. (2025), who observed a T_5_% of 325 °C for PLA and 320 °C for rPLA, with corresponding T_max_ values of 366 °C and 360 °C, respectively [3]. This moderate shift also agreed with recent studies reporting that reprocessing can reduce the molecular weight without necessarily causing drastic changes in the overall thermal response, although it may slightly displace characteristic degradation temperatures depending on the processing history and the matrix state [51].

The addition of ATBC produced the most pronounced change in the onset degradation temperature at 5% mass loss, since the T_5_% decreased to 245.0 °C (PLA–ATBC) and 241.0 °C (rPLA–ATBC), while the T_max_ remained relatively close to the PLA range (364.6–358.4 °C). This behavior was interpreted as typical of citrate plasticization: the decrease in the T_5_% was largely governed by early volatilization and/or degradation of the plasticizer and by increased free volume and segmental mobility, mainly because ATBC possesses oligomeric chains with lower molecular weights than PLA and rPLA, which degrade at lower temperatures. This effect has been documented for PLA formulations containing citrate-type plasticizers, where the onset degradation temperature measured at 5% mass loss shifts to lower temperatures [38,52].

For the unplasticized composites, RH produced only minor effects: rPLA–1% RH exhibited values close to those of rPLA (T_5_% = 319.4 °C; T_max_ = 368.2 °C), whereas rPLA–3% RH showed a moderate decrease in the T_5_% (315.7 °C) while maintaining an essentially stable T_max_ (368.9 °C). These results suggest that, at these loading levels, the lignocellulosic reinforcement did not dominate the primary degradation event, although it may have contributed to slight variations associated with microstructural differences and matrix–particle interactions.

In contrast, biochar (RHB) exhibited a loading-dependent response: at 1 wt.%, rPLA–1% RHB increased the T_5_% to 325.2 °C, suggesting a mild stabilizing effect, whereas at 3 wt.%, the stability decreased (T_5_% = 312.7 °C; T_max_ = 365.2 °C). This trend was consistent with reports on biochar-filled systems where higher loadings may introduce catalytic effects (e.g., ash/mineral species and reactive surface sites), agglomeration, and microdefects that accelerate PLA degradation and shift the T_max_ to lower temperatures [22,53].

Finally, in the ATBC-filled composites, the T_5_% remained low (≈242–255 °C), reinforcing that the onset was controlled by the plasticizer component, while the T_max_ tended to decrease when ATBC was combined with higher RHB contents (e.g., T_max_ = 355.8 °C for rPLA–3%RHB–ATBC), suggesting an additional reduction in thermal stability in scenarios where high melt mobility coexisted with potential biochar-related catalytic effects at higher loading [22,52].

### 3.7. Water Contact Angle

**Figure 11:**
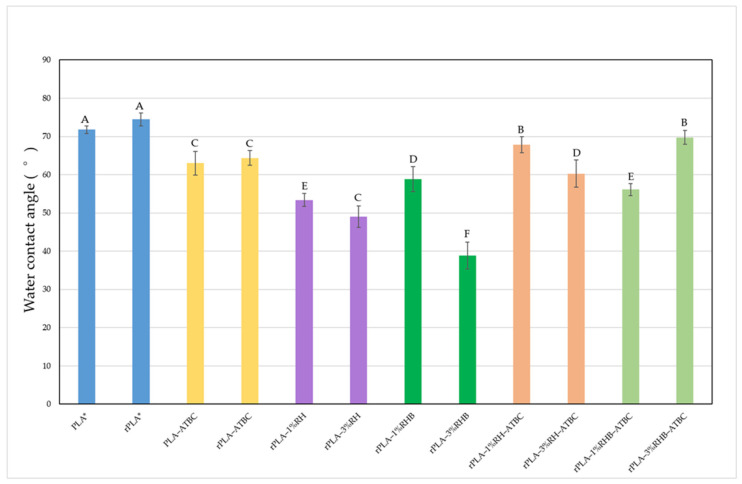
Static water contact angle of PLA, rPLA and rPLA composites films. Different letters (A–F) indicate statistically significant differences among the formulations (*p* < 0.05). * The WCA results corresponding to PLA and rPLA were taken from the previous work of Gonzalez-Serrud et al. (2026) [16] and are included here for comparison purposes.

The surface wettability of the developed films was assessed by static water contact angle (WCA) measurements in accordance with ASTM D5946-17. As shown in Figure 11, the WCA values exhibited pronounced differences in surface hydrophilicity/hydrophobicity depending on the polymer matrix and the type of filler incorporated.

The contact angle results indicated that PLA exhibited moderately hydrophobic behavior, with a WCA of 71.8 ± 1.0° [16], which fell within the typical range reported for untreated PLA films (71–74.6°) [3,54,55]. rPLA showed a small increase (74.5 ± 1.7°), consistent with slight changes in surface chemistry and morphology after reprocessing [16]. ATBC reduced the WCA in both neat PLA (PLA–ATBC: 63.0 ± 3.1°) and the reprocessed PLA matrix (rPLA–ATBC: 64.4 ± 1.9°), indicating a shift toward higher surface wettability, which may be attributed to plasticizer-induced increases in chain mobility and the preferential orientation of more polar citrate-rich domains at the surface [14,15,37].

The incorporation of RH markedly decreased the WCA (rPLA–1%RH: 53.4 ± 1.7°; rPLA–3% RH: 49.0 ± 2.8°), which was consistent with the hydroxyl-rich lignocellulosic surface promoting water affinity and capillary wetting [56]. The RHB presence also reduced the WCA relative to the neat matrices (rPLA–1% RHB: 58.9 ± 3.3°), and the strongest decrease in the dataset was observed at 3 wt.% RHB (38.9 ± 3.5°), suggesting that at higher loadings, the biochar surface and/or its microporosity increased the effective wetting by enhancing liquid spreading and water retention at the surface [57].

When ATBC was combined with fillers, the response depended on the filler type and loading. rPLA–1% RH–ATBC and rPLA–3% RH–ATBC displayed higher WCA values than their non-plasticized counterparts (67.9 ± 2.1° and 60.3 ± 3.6°, respectively), which suggested that ATBC partially offsets the hydrophilicity introduced by RH, likely by improving matrix wetting and reducing exposed hydrophilic sites at the surface. For RHB, ATBC led to a low WCA at 1 wt.% (56.1 ± 1.5°) but an unexpected increase at 3 wt.% (69.8 ± 1.8°), which suggested a shift in surface composition and topography, potentially associated with plasticizer-driven surface segregation and a redistribution of exposed RHB domains.

Overall, the WCA data indicated that RH consistently increased surface hydrophilicity with loading, whereas RHB exhibited a stronger and more formulation-sensitive effect, and ATBC tended to increase the wettability in neat matrices while modulating the apparent surface polarity of the composites depending on the filler content.

### 3.8. Water Vapor Transmission Rate

**Figure 12:**
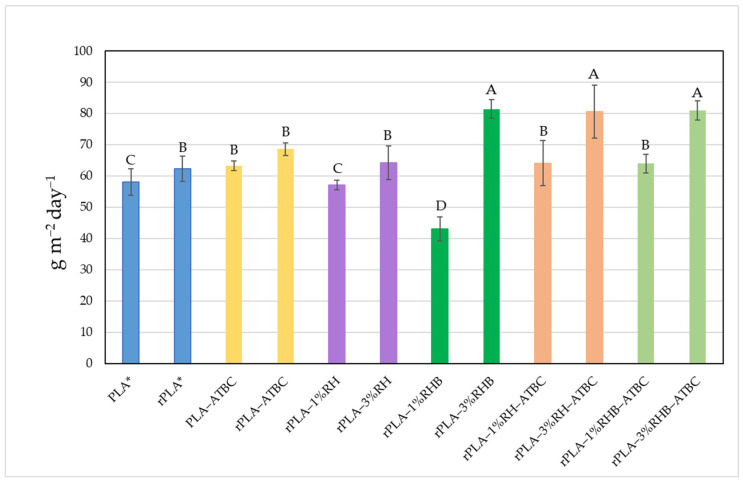
The results from the WVTR. Different letters (A–D) indicate statistically significant differences among the formulations (*p* < 0.05). * The WVTR results corresponding to PLA and rPLA were taken from the previous work of Gonzalez-Serrud et al. (2026) [16] and are included here for comparison purposes.

Figure 12 summarizes the water vapor transmission rate (WVTR) results, which quantify the water vapor permeability of PLA films and their composite counterparts reinforced with rice husk (RH) or rice husk biochar (RHB), with and without ATBC plasticization.

The WVTR results of Gonzalez-Serrud et al. (2026) [16] indicated that the permeability was strongly governed by the combined effects of reprocessing, plasticization, and filler type/loading. PLA exhibited a WVTR of 58.1 ± 4.2 g·m^−2^·day^−1^, whereas rPLA increased to 62.4 ± 4.1 g·m^−2^·day^−1^, which suggested a slight loss of barrier performance after reprocessing [16]. This trend was consistent with chain scission and a higher fraction of mobile amorphous segments and chain ends, which typically facilitated water vapor diffusion [3,7]. The incorporation of ATBC further increased the WVTR in both matrices (PLA–ATBC: 63.3 ± 3.3; rPLA–ATBC: 65.6 ± 3.0 g·m^−2^·day^−1^), as expected for plasticized systems, since increased free volume and segmental mobility generally enhance vapor transport and could also promote microvoid-assisted diffusion if plasticizer redistribution occurs during conditioning [14,37].

The fillers produced distinct responses depending on their chemistry and loading. At 1 wt.% RH, the WVTR slightly decreased relative to rPLA (57.2 ± 1.6 g·m^−2^·day^−1^), which suggested that, at low loading, the filler increased the tortuosity and promoted a microstructure that partially offset the hydrophilic character of the lignocellulosic particles when dispersion was adequate. However, increasing RH to 3 wt.% raised the WVTR to 64.3 ± 5.4 g·m^−2^·day^−1^, and the combination with ATBC led to a pronounced increase (80.6 ± 6.0 g·m^−2^·day^−1^) [15]. These results indicated that higher RH contents likely generated more continuous hydrophilic domains and interfacial microgaps that provided preferential pathways for moisture transport, an effect that was amplified by the higher matrix mobility induced by ATBC.

Rice husk biochar showed the most favorable barrier effect at low loading. The 1 wt.% RHB formulation exhibited the lowest WVTR in the dataset (43.1 ± 3.9 g·m^−2^·day^−1^), corresponding to an approximately 31% reduction relative to rPLA. This behavior was consistent with the more carbonized and comparatively less polar character of RHB, which could have increased diffusion tortuosity and reduced sorption-driven transport. In contrast, at 3 wt.% RHB, the WVTR increased sharply (78.0 ± 5.2 g·m^−2^·day^−1^) and remained high with ATBC (81.0 ± 2.2 g·m^−2^·day^−1^), suggesting that, at higher loadings, the porous nature of biochar, possible agglomeration, and interfacial void formation dominated the barrier response by creating defect-assisted diffusion pathways [57].

Among the ATBC-plasticized composites, rPLA–1%RHB–ATBC exhibited the lowest WVTR value, 64.0 ± 2.10 g·m^−2^·day^−1^, indicating the best barrier performance within this group. This behavior suggests that a low RHB loading partially compensates for the increase in free volume and segmental mobility caused by ATBC, probably by increasing diffusion tortuosity and reducing moisture sorption due to the more carbonized and less polar character of biochar. However, at 3 wt.% RHB, the WVTR increased markedly, suggesting that particle agglomeration, the interfacial voids, and the porous structure of biochar became dominant, creating preferential pathways for water vapor transport [58]. Thus, the WVTR results indicate that low filler loadings, especially 1 wt.% RHB, can improve the barrier response through a tortuosity-dominated mechanism, whereas higher filler loadings shift the system toward a permeability-dominated behavior controlled by hydrophilic domains, interfacial microgaps, porosity, and increased chain mobility.

### 3.9. Disintegration Under Composting Conditions

**Figure 13:**
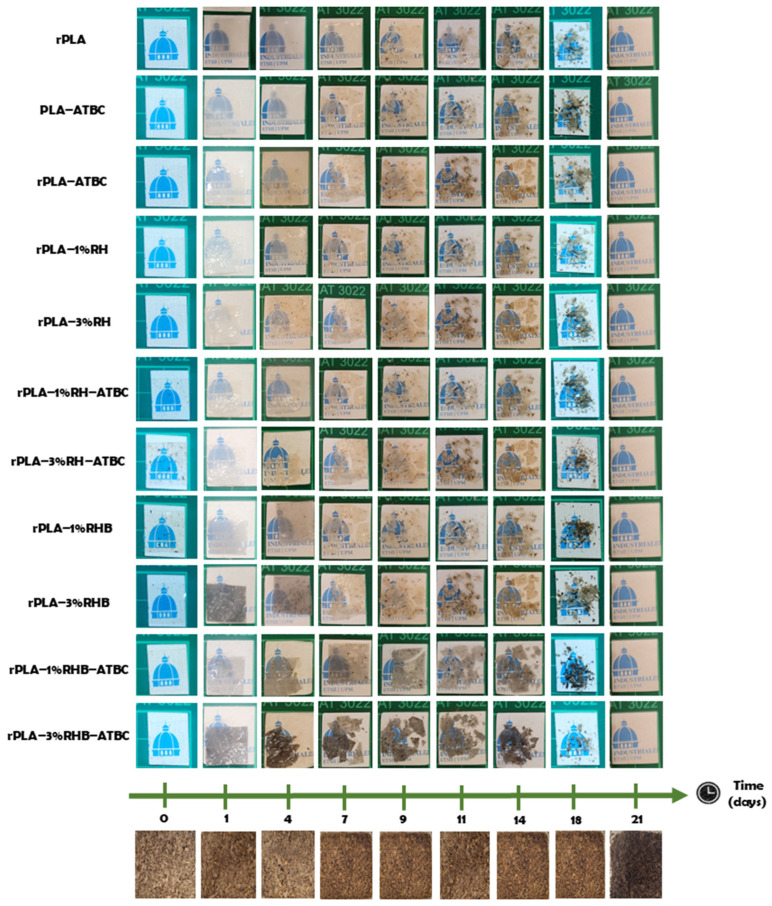
Evolution of the visual properties of PLA and rPLA films reinforced with RH and RHB during compost incubation. Photographs corresponding to rPLA films were adapted from [21].

**Figure 14:**
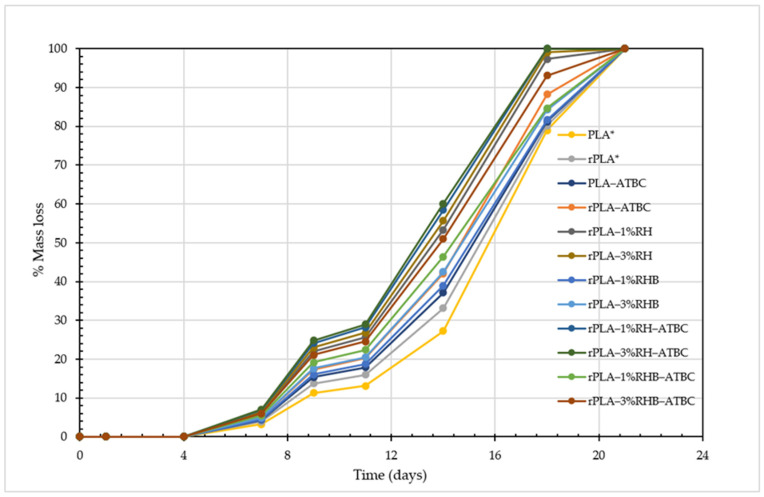
Disintegration curves under composting conditions. * The disintegration results corresponding to PLA and rPLA were taken from the previous work of Gonzalez-Serrud et al. (2026) [16] and are included here for comparison purposes.

The disintegration performance of the composites under laboratory-simulated composting was evaluated by monitoring the time-dependent mass loss of the buried specimens, following the UNE EN-ISO 20200 [3]. Figure 13 shows the evolution of the films’ appearance during compost incubation for the PLA and rPLA formulations reinforced with RH and RHB, and Figure 14 presents the corresponding disintegration curves as a function of composting time.

On day 1, the films with and without plasticizers exhibited a noticeable reduction in transparency, indicating the onset of hydrolytic processes and microbial activity, which are promoted by the elevated incubation temperature (58 °C) and the high moisture content of the composting medium [3]. This early loss of transparency is consistent with rapid water uptake by the hydrophilic domains within the films; such an effect may be further intensified in composites containing natural particulates (rice husk and rice husk biochar), which can increase the overall surface polarity and facilitate moisture penetration. As degradation progressed, the specimens became increasingly opaque [40,59].

The progressive opacity increase can be linked to both the physical and chemical deterioration of the polymer matrix, accompanied by a gradual loss of structural integrity and the appearance of visible signs of microbial colonization and enzyme-driven degradation [60]. By day 21, the films were fully disintegrated and incorporated into the compost substrate. At this stage, transparency was completely lost as the materials fragmented into smaller pieces and underwent advanced molecular breakdown, resulting in effective integration with the surrounding organic matter [61].

Disintegration under simulating composting conditions revealed an initial phase in which no net mass loss was recorded up to day 4; however, this does not indicate the absence of degradation, since this stage is dominated by swelling due to water absorption, which can mask the actual material loss by partially offsetting it with moisture-induced weight gain. From day 7 onward, a clearly measurable mass decrease was observed, indicating the onset of a fragmentation-driven stage and accelerated disintegration [5,61].

Compared with PLA, rPLA exhibited faster disintegration during the intermediate stage (e.g., 33.1% vs. 27.3% at day 14) [16], which is consistent with its lower molecular weight and the higher concentration of chain ends typically generated after an additional melt-processing cycle, thereby increasing its susceptibility to hydrolysis [3].

The incorporation of 1 wt.% RH or RHB further accelerated the disintegration relative to rPLA (day 14: 38.9% for RH and 37.1% for RHB vs. 33.1% for rPLA), in agreement with the development of moisture-transport pathways at the filler–matrix interface and, in the case of RH, a more hydrophilic surface chemistry that can facilitate water diffusion compared with the more carbonized RHB [36].

ATBC plasticization systematically increased the disintegration kinetics in both the unfilled and composite systems; for instance, PLA increased from 27.3% to 42.1% at day 14 with ATBC, and rPLA from 33.1% to 42.5%. This trend is expected because plasticization increases free volume and segmental mobility, promoting water diffusion and hydrolytic chain scission, while potential plasticizer redistribution may contribute to microvoid formation and faster fragmentation [15].

Finally, it should be noted that ISO 20200 primarily quantifies physical disintegration (mass loss/fragmentation) under controlled conditions and, by itself, does not confirm complete biodegradation or mineralization.

### 3.10. Practical Implications, Advantages, and Limitations of the Developed Films

Table 6 summarizes the application-oriented implications of the developed rPLA-based composite films by comparing the main advantages, limitations, and realistic uses associated with each formulation component. This comparison helps to identify the most relevant trade-offs between circularity, flexibility, barrier behavior, composting disintegration, and practical applicability.

From an application-oriented perspective, the developed rPLA-based composite films should be considered tunable short-life compostable materials rather than direct high-performance substitutes for all PLA-based films. Their main contribution is the simultaneous valorization of mechanically reprocessed PLA (rPLA), representing a closed-loop route for industrial PLA scraps, and rice-derived residues incorporated as untreated rice husk (RH) or rice husk biochar (RHB). This approach is consistent with recent studies showing that reprocessed PLA can be reformulated into functional films when plasticization and low-content particulate reinforcement are properly combined [9,17].

Compared with the PLA homopolymer, the developed materials show both advantages and trade-offs. Neat PLA generally provides higher tensile strength and stiffness, but its brittleness limits its use in flexible-film applications. The incorporation of ATBC increases chain mobility, improves melt flowability, and enhances film handling, in agreement with the reported role of citrate-based plasticizers in reducing PLA brittleness and increasing ductility [38,39,65]. However, plasticization also reduces tensile strength and modulus and may increase free volume, promoting moisture transport or plasticizer redistribution. Therefore, these films are more suitable for applications where flexibility, processability, and controlled disintegration are more important than maximum mechanical performance.

RH and RHB provide additional formulation tools to tune the properties of rPLA-based films. RH is an abundant lignocellulosic residue with low cost and high availability, whereas RHB is a more carbonized, thermally stable, and less polar material obtained by the pyrolysis of the low-cost RH. Previous studies have shown that rice-derived fillers can modify stiffness, crystallization, water uptake, and degradation-related behavior depending on their chemistry, loading, dispersion, and interfacial adhesion [18,36]. In this study, the RH-containing films are more appropriate when increased hydrophilicity and faster disintegration are desired, while the low-RHB formulations are more promising when improved water vapor barrier performance is required.

The best functional advantage was observed for the low-loading RHB formulation, which improved the water vapor barrier behavior. This effect can be associated with the ability of well-dispersed carbonaceous particles to increase the tortuosity of the diffusion pathway and reduce sorption-driven transport. Nevertheless, this benefit should not be generalized to higher concentrations. Previous PLA/biochar studies have reported that low biochar contents may improve selected properties, whereas higher loadings can promote agglomeration, interfacial defects, and deterioration of mechanical or barrier performance [17,24]. In this work, the higher filler contents and ATBC tended to increase the WVTR, probably because the tortuosity effect was overcome by filler-related defects, porosity-associated pathways, and higher matrix mobility.

The main limitation of the RH-containing films is their higher affinity for water due to their hydroxyl-rich lignocellulosic surface. This feature can accelerate composting disintegration by promoting water uptake and hydrolytic attack on PLA, but it can also be detrimental for applications requiring high moisture resistance. Natural fibers and lignocellulosic particles may act as nucleating agents and facilitate fragmentation, although they can also increase hydrolysis, interfacial debonding, and premature mechanical failure when filler–matrix adhesion is insufficient [40,59].

Accordingly, the most realistic applications for these films are agricultural and non-structural short-life uses, such as compostable soil-covering films, nursery or seedling-related flexible sheets, short-term mulch-like films, compostable collection bags, and dry non-food packaging where moderate water vapor barrier performance is acceptable. This positioning agrees with the development of biodegradable mulch and soil-covering films as alternatives to conventional polyethylene films, potentially reducing removal and disposal after use [63,66,67]. However, laboratory disintegration under composting conditions should not be interpreted as complete biodegradation or mineralization under real soil environments. Future work should evaluate wider filler contents, aging, soil-burial behavior, field performance, plasticizer migration, ecotoxicity, and complete biodegradation to confirm their practical viability.

## 4. Conclusions

This work demonstrated that mechanically reprocessed PLA (rPLA) could be converted into circular biocomposite films by the combination of plasticization with ATBC and reinforcement with rice-husk-derived materials (RH and RHB), while retaining full end-of-life disintegration under composting conditions. Reprocessing increased melt flowability and reduced molecular indicators, suggesting chain scission, while producing a moderate decline in tensile performance relative to virgin PLA; nevertheless, rPLA remained a viable matrix for formulation-driven property tuning.

ATBC was the dominant variable controlling processability and ductility, markedly increasing the MFI and the elongation at break while reducing the tensile strength and the stiffness, as expected for plasticized PLA systems. RH and RHB primarily acted as nucleating agents, increasing the crystallinity without significantly shifting the T_m_, whereas ATBC reduced the T_g_ and modified the cold-crystallization behavior, with filler-dependent crystallinity outcomes in the plasticized composites. The thermogravimetric trends showed a slight stability decrease after reprocessing and a pronounced reduction in the T_5%_ upon ATBC addition, consistent with early mass-loss events associated with citrate plasticization, while the main degradation peak remained comparatively stable except when combined with a higher RHB content.

The functional properties were strongly formulation-dependent: a low RHB content (1 wt.%) provided the best water vapor barrier response, whereas higher filler loadings and ATBC generally increased the WVTR. The surface wettability increased with the RH loading and varied with the RHB and ATBC combinations, evidencing that the surface chemistry and the phase distribution governed the apparent hydrophilicity. Finally, all the films reached 100% disintegration by day 21, and higher filler contents accelerated the disintegration kinetics, with the rPLA–3% RH–ATBC and rPLA–3% RHB–ATBC formulations reaching nearly complete disintegration by day 18. Overall, the results established practical formulation pathways to tune the processing, the physicochemical performance, and the disintegration rate of rPLA films, supporting their development as sustainable polymer materials aligned with circular-economy principles.

In conclusion, the ATBC-plasticized formulations exhibited higher surface wettability, increased the WVTR, and had faster disintegration kinetics compared with the unplasticized systems. These effects are associated with the effective plasticizing action of ATBC, which increases free volume and chain mobility in the PLA/rPLA matrix, facilitating water diffusion and hydrolytic degradation. As a result, ATBC can enhance water vapor transport and accelerate physical fragmentation, explaining the combined increase in wettability, permeability, and disintegration rate observed in the plasticized films.

## Figures and Tables

**Table 1:** Film formulations developed in this study. Compositions are expressed in wt.%.

Sample (Film)	Matrix	RH (wt.%)	RHB (wt.%)	ATBC (wt.%)
PLA	PLA	-	-	-
rPLA	PLA	-	-	-
PLA–ATBC	rPLA	-	-	15
rPLA–ATBC	rPLA	-	-	15
rPLA–1%RH	rPLA	1	-	-
rPLA–3%RH	rPLA	3	-	-
rPLA–1%RHB	rPLA	1	-	15
rPLA–3%RHB	rPLA	3	-	15
rPLA–1%RH–ATBC	rPLA	-	1	-
rPLA–3%RH–ATBC	rPLA	-	3	-
rPLA–1%RHB–ATBC	rPLA	-	1	15
rPLA–3%RHB–ATBC	rPLA	-	3	15

**Table 2:** The thermal properties of poly(lactic acid) (PLA) and PLA composites reprocessed (rPLAs) with rice husk (RH) and carbonized rice husk (RHB) were evaluated, including measurements of glass transition temperature (T_g_), cold crystallization temperature (T_cc_), melting temperature (T_m_), cold crystallization enthalpy (ΔH_cc_), enthalpy of fusion (ΔH_m_), and degree of crystallinity (X_c_). * The DSC data corresponding to PLA and rPLA were obtained from the previous study reported by [16] and are included here for comparison purposes.

Sample (Film)	T_g_ (°C)	T_cc_ (°C)	T_m_ (°C)	ΔH_cc_ (J/g)	ΔH_m_ (J/g)	X_c_ (%)
* PLA	59.0	119.9	149.7	26.0	33.4	8.0
* rPLA	59.2	115.2	148.9	29.1	38.4	10.0
PLA–ATBC	39.1	117.5	150.7	27.5	31.4	4.9
rPLA–ATBC	37.9	95.8	149.8	28.0	28.2	0.2
rPLA–1%RH	59.1	115.3	149.0	36.9	60.4	25.5
rPLA–3%RH	59.1	117.8	149.9	38.1	63.6	28.2
rPLA–1%RHB	59.1	116.4	149.6	34.6	61.0	28.6
rPLA–3%RHB	59.1	117.4	149.8	37.2	58.2	23.3
rPLA–1%RH–ATBC	39.2	97.0	150.6	28.9	29.6	0.9
rPLA–3%RH–ATBC	37.3	96.5	150.2	27.3	31.1	5.0
rPLA–1%RHB–ATBC	43.9	106.7	150.4	39.4	63.5	30.8
rPLA–3%RHB–ATBC	40.8	107.0	150.0	37.3	60.5	30.4

**Table 3:** Thermogravimetric parameters of rice husk (RH).

Sample (Film)	T_5_% (°C)	T_10_% (°C)	DTG Peak 1 (°C)	DTG Peak 2 (°C)	DTG Peak 3 (°C)	Final Residue (%)
RH	234.3	277.9	346.2	426.1	508.7	15.8

**Table 4:** Thermogravimetric parameters of rice husk biochar (RHB).

Sample (Film)	T_5_% (°C)	T_10_% (°C)	DTG Peak 1 (°C)	DTG Peak 2 (°C)	Final Residue (%)
RHB	335.6	422.7	359.2	613.0	33.7

**Table 5:** Comparison of decomposition temperatures and mass loss in different PLA film samples. * The TGA data corresponding to PLA and rPLA were obtained from the previous study reported by [16] and are included here for comparison purposes.

Sample (Film)	T_5_% (°C)	T_max_ (°C)
* PLA	323.4	371.2
* rPLA	318.2	364.7
PLA–ATBC	245.0	364.6
rPLA–ATBC	241.0	358.4
rPLA–1%RH	319.4	368.2
rPLA–3% RH	315.7	368.9
rPLA–1% RHB	325.2	369.6
rPLA–3% RHB	312.7	365.2
rPLA–1%RH–ATBC	249.5	367.2
rPLA–3%RH–ATBC	245.8	360.3
rPLA–1%RHB–ATBC	255.3	363.7
rPLA–3%RHB–ATBC	242.7	355.8

**Table 6:** Practical relevance, advantages, and limitations of the developed rPLA/RH and rPLA/RHB composite films.

Material/Design Aspect	Main Advantage	Main Limitation	Most Realistic Application Relevance	References
rPLA matrix	Valorizes industrial PLA scraps and reduces the dependence on virgin PLA.	Reprocessing can promote chain scission, reducing molecular weight and ductility.	Circular short-life films and closed-loop industrial-waste valorization.	[3,7]
ATBC plasticization	Plasticizer improves flexibility, melt flowability, and film handling.	Reduces tensile strength and stiffness and may increase moisture transport.	Flexible compostable films where ductility is prioritized over high strength.	[44,62]
RH addition	Uses an abundant lignocellulosic residue and increases hydrophilicity and disintegration tendency.	May increase WVTR, water uptake, and interfacial debonding at higher loading.	Soil-contact or compostable applications requiring fast disintegration.	[18,21]
RHB addition	Provides a carbon-rich, thermally stable filler; low RHB loading may improve WVTR through tortuosity.	Higher loading may promote agglomeration, porosity-related transport, or interfacial defects.	Films requiring moderate barrier improvement while retaining compostable disintegration.	[17,24]
Composting behavior	Fast physical disintegration under laboratory composting conditions.	Laboratory disintegration does not demonstrate complete biodegradation or mineralization.	Short-life compostable items, provided end-of-life conditions are controlled.	[40,59,63]
Practical use scenario	Combines rPLA valorization, agro-residue utilization, flexibility, and tunable water-related behavior.	Requires field, migration, aging, ecotoxicity, and full biodegradation studies before industrial claims.	Agricultural soil-covering films, nursery sheets, compostable bags, and dry non-food packaging.	[63,64]

## Data Availability

The original contributions presented in this study are included in the article. Further inquiries can be directed to the corresponding authors.
